# Mutants of *Lotus japonicus* deficient in flavonoid biosynthesis

**DOI:** 10.1007/s10265-021-01258-8

**Published:** 2021-02-11

**Authors:** Toshio Aoki, Masayoshi Kawaguchi, Haruko Imaizumi-Anraku, Shoichiro Akao, Shin-ichi Ayabe, Tomoyoshi Akashi

**Affiliations:** 1 Department of Applied Biological Sciences, Nihon University, Fujisawa, Kanagawa 252-0880 Japan; 2 Division of Symbiotic Systems, National Institute for Basic Biology, Okazaki, Aichi 444-8585 Japan; 3 Institute of Agrobiological Sciences, National Agriculture and Food Research Organization, Tsukuba, Ibaraki 305-8634 Japan

**Keywords:** Anthocyanin, Condensed tannin, *Lotus japonicus* (regel) K.Larsen, Mutant, Proanthocyanidin, *VIRIDICAULIS*

## Abstract

Spatiotemporal features of anthocyanin accumulation in a model legume *Lotus japonicus* (Regel) K.Larsen were elucidated to develop criteria for the genetic analysis of flavonoid biosynthesis. Artificial mutants and wild accessions, with lower anthocyanin accumulation in the stem than the standard wild type (B-129 ‘Gifu’), were obtained by ethyl methanesulfonate (EMS) mutagenesis and from a collection of wild-grown variants, respectively. The loci responsible for the green stem of the mutants were named as *VIRIDICAULIS* (*VIC*). Genetic and chemical analysis identified two loci, namely, *VIC1* and *VIC2*, required for the production of both anthocyanins and proanthocyanidins (condensed tannins), and two loci, namely, *VIC3* and *VIC4*, required for the steps specific to anthocyanin biosynthesis. A mutation in *VIC5* significantly reduced the anthocyanin accumulation. These mutants will serve as a useful system for examining the effects of anthocyanins and proanthocyanidins on the interactions with herbivorous pests, pathogenic microorganisms and nitrogen-fixing symbiotic bacteria, *Mesorhizobium loti*.

## Introduction

Higher plants produce diverse flavonoid metabolites involved in a number of plant functions (Dixon and Steele 1999; Winkel-Shirley 2001), such as pigmentation, pollen fertility (van der Meer et al. 1992; Ylstra et al. 1994), auxin transport (Jacobs and Rubery 1988; Peer and Murphy 2007), hydrogen peroxide scavenging (Morimoto et al. 1998; Yamasaki et al. 1997) and protection against harmful ultraviolet (UV) radiation (Chappell and Hahlbrock 1984; Li et al. 1993; Stapleton and Walbot 1994). Furthermore, flavonoids in leguminous plants play important roles (Aoki et al. 2000) in nodule formation as signal molecules (Franssen et al. 1992; Long 1996) or in defence responses as antimicrobial phytoalexins (Dixon and Paiva 1995). Flavonoids have also been implicated in root and nodule organogenesis (Charrier et al. 1998; Hassan and Mathesius 2012; Mathesius 2001; Mathesius et al. 1998; Spaink 1998; Subramanian et al. 2006; Wasson et al. 2006; Zhang et al. 2009) and architectural phenotypes (Buer and Djordjevic 2009).

Anthocyanins (blue–red pigments) are one of the principal classes of flavonoids. Pigments in reproductive organs serve as attractants for pollinators and seed dispersers. The biosynthesis of anthocyanins has been extensively studied, and the biosynthetic enzymes and genes involved have been characterized (Hrazdina 1982; Mulder-Krieger and Verpoorte 1994). Anthocyanin biosynthesis in *Arabidopsis thaliana*, maize kernels, morning glory and petunia and snapdragon flowers represents a good system for genetic studies, and several structural and regulatory genes in this pathway have been identified using these plant species (Forkmann 1994; Holton and Cornish 1995; Park et al. 2018; Sharma et al. 2011; Winkel-Shirley et al. 1995).

**Fig. 1:**
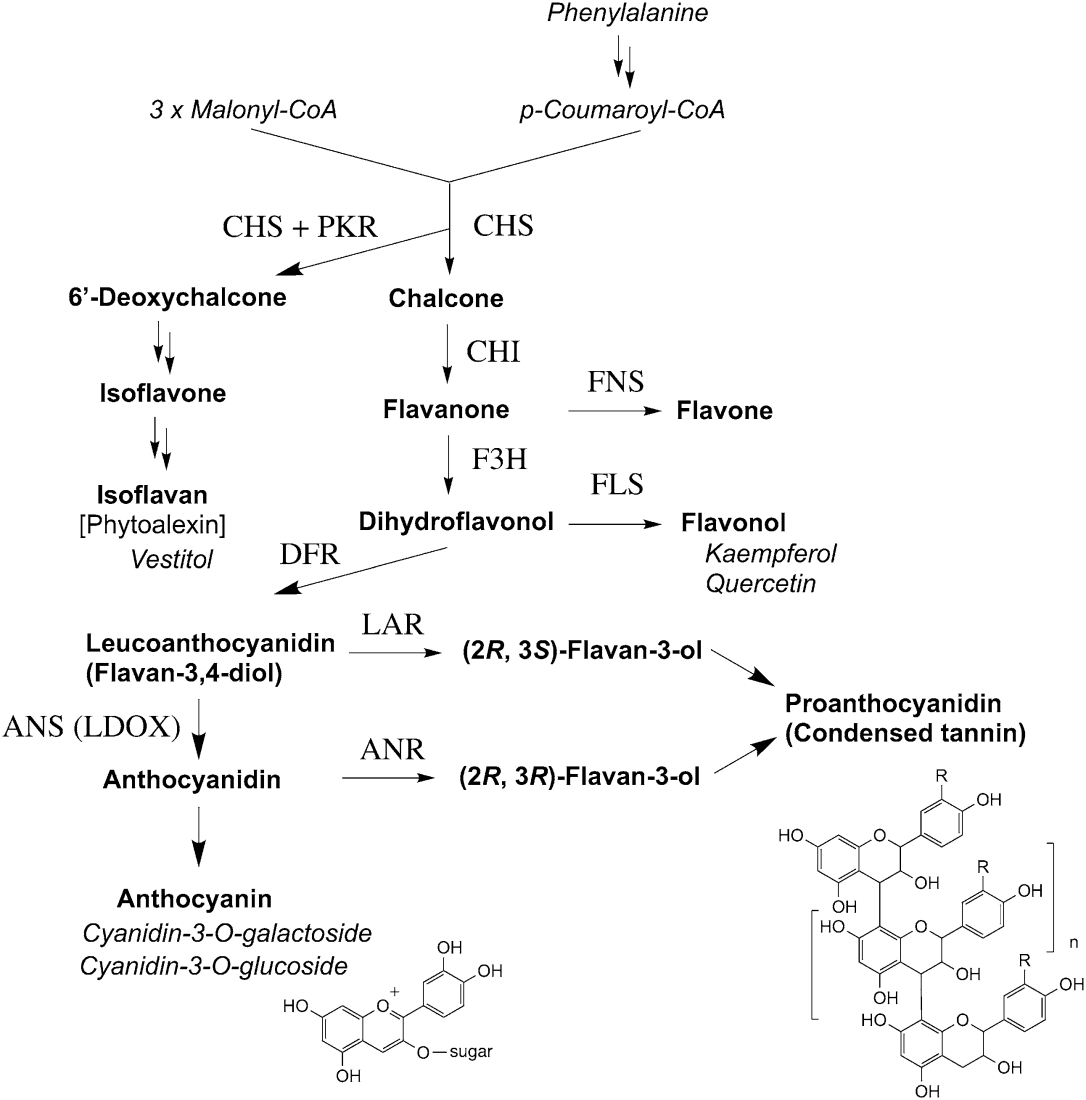
Flavonoid biosynthetic pathway in leguminous plants. Names of flavonoid skeletons and individual compounds are shown in bold and italics, respectively. *ANR* anthocyanidin reductase, *ANS* anthocyanidin synthase, *CHS* chalcone synthase, *CHI* chalcone isomerase, *DFR* dihydroflavonol 4-reductase, *F3H* flavanone 3β-hydroxylase, *FNS* flavone synthase, *FLS* flavonol synthase, *LAR* leucoanthocyanidin reductase, *LDOX* leucoanthocyanidin dioxygenase, *PKR* polyketide reductase

Proanthocyanidins, also known as condensed tannins, represent another class of flavonoid metabolites (Haslam 1998). These are flavonoid oligomers biosynthesized by a branch of the flavonoid pathway (Fig. 1) and have been shown to affect the resistance against herbivorous insects (Chan et al. 1978) and the interaction with nitrogen-fixing rhizobia (Jones et al. 1987; Punkhurst and Jones 1979). Bird’s-foot trefoil (*Lotus corniculatus* L.), a leguminous forage crop, is characterized by the accumulation of important anti-bloating agents, proanthocyanidins (Jones and Lyttleton 1971). Therefore, biochemical and molecular studies on the proanthocyanidin pathway have been performed using *L. corniculatus* and *Lotus japonicus* (Regel) K.Larsen (Bavage et al. 1997; Escaray et al. 2017; Gruber et al. 2008; Lees 1986; Morris and Robbins 1992; Morris et al. 1993; Robbins et al. 1998; Sivakumaran et al. 2006; Skadhauge et al. 1997), the latter of which has been used as a model legume for molecular genetics and genomics studies (Handberg and Stougaard 1992; Jiang and Gresshoff 1997; Sato et al. 2001, 2008).

Although some flavonoid biosynthetic genes are regulated by developmental cues, the biosynthesis of anthocyanins and other flavonoids is also modulated by environmental conditions (Hrazdina 1982; McClure 1975), such as light (Chappell and Hahlbrock 1984; McNellis and Deng 1995), low temperature (Christie et al. 1994), nutrient availability (Coronado et al. 1995), wounding and pathogen attack (Dixon and Paiva 1995). Sensing the external stimuli and transducing these signals are critical for plant survival; however, the molecular bases of these processes remain to be elucidated. Investigation of the regulation of the flavonoid biosynthesis will advance our understanding of the plant response to environmental stimuli.

In this study, we investigated the effect of environmental conditions on anthocyanin accumulation in the stem of *L. japonicus* plants by performing mutant screening and genetic analysis. First, we investigated the characteristics of anthocyanin accumulation in *L. japonicus* B-129 ‘Gifu’ to establish the criteria for screening and evaluating mutants. Next, we isolated and characterized artificial mutants and wild accessions deficient in anthocyanin accumulation, MG-13 ‘Arasaki’, MG-17 ‘Mishima’ and MG-20 ‘Miyakojima’. Genetic analysis using the mutants and wild accessions identified at least five loci essential for the proper accumulation of anthocyanins in the *L. japonicus* stems. Since the biosynthetic pathway of anthocyanins is linked to that of flavones, flavonols and proanthocyanidins (Fig. 1), the anthocyanin mutants included mutants with low proanthocyanidin accumulation. Various flavonoid-accumulating mutants identified in this study and wild accessions should serve as useful resources for elucidating the specific biological roles of flavonoids in legumes.

## Materials and methods

### Plant materials and screening for anthocyanin-deficient mutants

Seeds of *L. japonicus* accession B-129 ‘Gifu’ (Grant et al. 1962; Handberg and Stougaard 1992) and MG-58 Nichinan were provided by Dr. J. Stougaard (Aarhus University, Denmark) and Dr. T. Shimada (Obihiro University of Agriculture and Veterinary Medicine, Japan), respectively. Other accessions were collected from several areas in Japan (Table 1). The growth conditions in the greenhouse and the ethyl methanesulfonate (EMS) mutagenesis protocol have been previously described (Imaizumi-Anraku et al. 1997). A total of 14,000 M_2_ seeds were sown in low-nutrient artificial soil, and seedlings were grown for 4–8 weeks. In the first screening, plants with no red pigmentation in the stem were selected and transferred to nutrient-rich soil. The putative mutants were then evaluated based on the stem at the flowering stage. Mutant lines were confirmed to be anthocyanin-deficient in the M_3_ generation and crossed with B-129 (wild type). F_2_ plants were grown in low-nutrient artificial soil for 6–8 weeks in a greenhouse, and the segregation of wild-type and anthocyanin-deficient phenotypes was observed. The mutants were grouped into classes and subclasses according to the phenotype. To perform complementation analysis, mutants within the same phenotypic subclass were crossed. To analyse anthocyanin accumulation in young plants in vitro, sterilized seeds were sown in a plastic pot (7 cm × 7 cm × 10 cm) containing B&D (Broughton and Dilworth 1971) agar (0.9%) medium supplemented with KNO_3_ (final concentration, 0.1 or 10 mM). The pots were sealed with Micropore Surgical Tape (3 M Health Care, St. Paul, MN, USA) to facilitate ventilation. Plants were grown in an incubator (Biotron LH 200, Nippon Medical and Chemical Instruments, Osaka, Japan) at 25 °C under a 16-h light/8-h dark cycle.

**Table 1:** Accessions of *L. japonicus* used in this study

Accession no.	Trivial name	Collection site
B-129	Gifu	See Grant et al. (1962)
MG-13	Arasaki	Yokosuka, Kanagawa. 35° 11′ N, 139° 36′ E
MG-15	Bishamon	Miura, Kanagawa. 35° 8′ N, 139° 39′ E
MG-17	Mishima	Mishima, Shizuoka. 35° 6′ N, 138° 56′ E
MG-20	Miyakojima	Gusukube (Miyakojima Island), Okinawa. 24° 43′ N, 125° 27′ E (Kawaguchi et al. 2001)
MG-58	Nichinan	Nichinan, Miyazaki. 31° 34′ N, 131° 24′ E

### Root nodule formation

*Mesorhizobium loti* strain Tono was isolated from a root nodule of *L. japonicus* accession MG-10 Tono. The isolated bacteria were used to inoculate *L. japonicus* B-129 seedlings grown on nitrogen-free B&D agar medium for 2 weeks, as previously described (Imaizumi-Anraku et al. 1997). The formation of root nodules was observed 1 month after inoculation.

### Anthocyanin quantification

Anthocyanins were extracted using 1% HCl in methanol and quantified by colorimetry according to the following equation:

$$Anthocyanin={A}_{530}-\left(0.25\times {A}_{657}\right)$$

### Flavonoid analysis

Stem segments [ca. 200 mg fresh weight (FW)] were extracted with five volumes of 80% (v/v) ethanol at 4 °C overnight. The soluble fraction was hydrolysed with 3 M HCl at 100 °C for 2 h and analysed by high-performance liquid chromatography (HPLC) equipped with a Shim-pack CLC-ODS column (150 mm length, 6 mm i.d; Shimadzu, Kyoto, Japan) using acetonitrile in water acidified with phosphoric acid (pH 3) as the mobile phase at a flow rate of 1 mL min^−1^ at 40 °C. Anthocyanidins were analysed with 16% acetonitrile (monitored at 550 nm), and non-anthocyanin flavonoid aglycones were separated with a linear gradient of 20–35% acetonitrile over 25 min (monitored at 236 nm). The insoluble fraction was treated with 5% (w/v) HCl in *n*-butanol at 90 °C for 60 min (Morris and Robbins 1992), yielding anthocyanidins derived from polymerized proanthocyanidins (condensed tannins). Anthocyanin glycosides were extracted with 3% acetic acid in ethanol at 4 °C overnight. The extract was partitioned with hexane to remove chlorophylls, and the acetic acid–ethanol layer was analyzed by HPLC using an XBridge C18 column (4.6 × 150 mm; Waters) at 40 °C a flow rate of 1.0 mL min^−1^ and a linear gradient elution for 20 min from 5 to 35% acetonitrile containing 0.5% trifluoroacetic acid. Quercetin, kaempferol, cyanidin, cyanidin-3-*O*-galactoside (idaein) and cyanidin-3-*O*-glucoside were identified by co-chromatography with authentic samples (Extrasynthese, Genay, France).

## Results

**Fig. 2:**
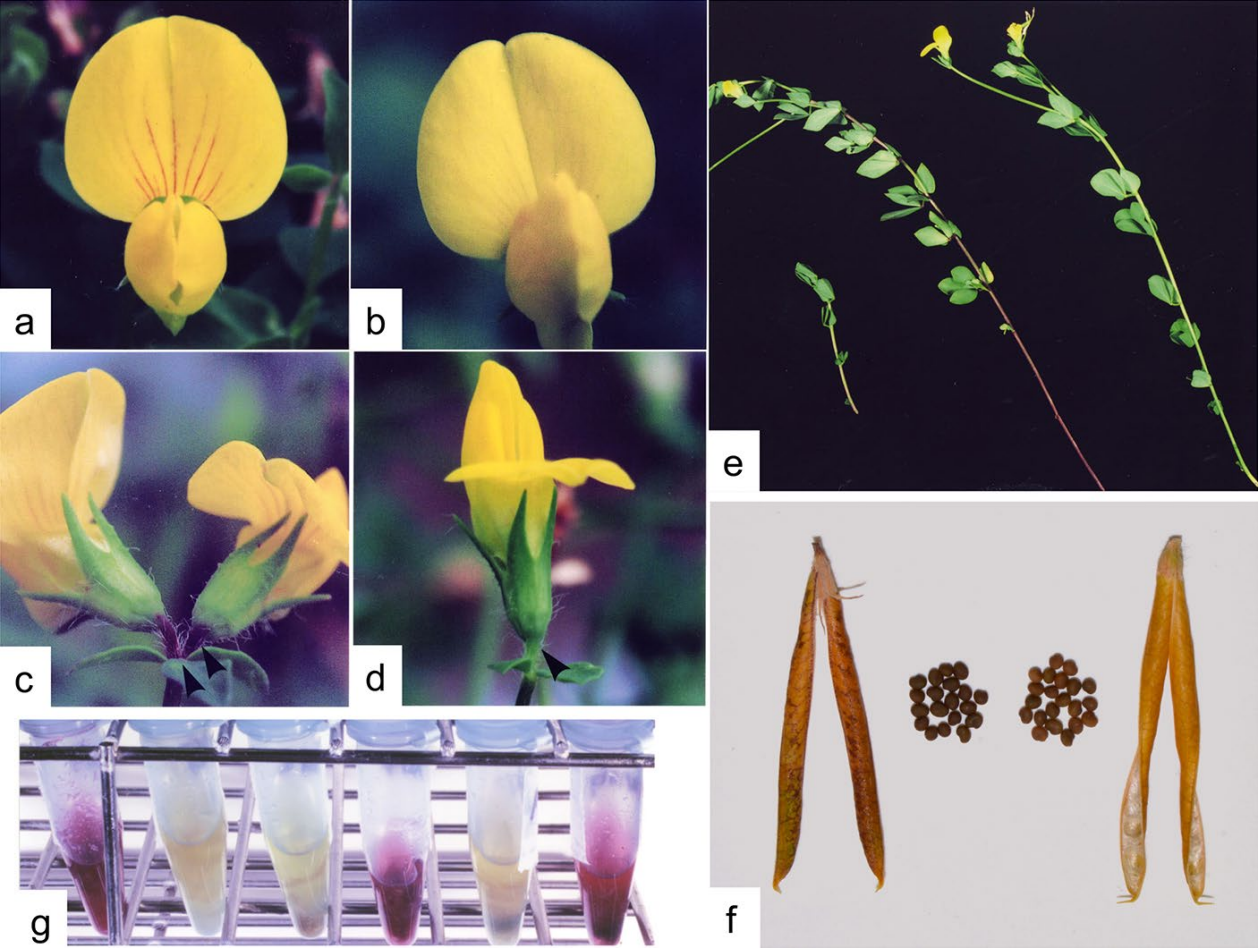
Anthocyanin accumulation and proanthocyanidin detection in *Lotus japonicus* B-129 ‘Gifu’ and *vic* mutants. **a** and **b** Petals of B-129 (**a**) and *vic3* (**b**). **c** and **d** Pedicels (arrowheads) of B-129 (**c**) and *vic3* (**d**). **e** B-129 (centre) and *vic1-1* (right) at the flowering stage. Left, 3-week old B-129 plant. **f** Pod and seeds of B-129 (left) and *vic1-2* (right). **g** Detection of polymerized proanthocyanidins (condensed tannins). The insoluble fraction of the stem was treated with 5% HCl in *n*-butanol at 90 °C for 60 min. Proanthocyanidins were converted to relevant anthocyanidins with red colour. Left to right: B-129, *vic1-1*, *vic2*, *vic3*, *vic1-2* and *vic4-1*

**Fig. 3:**
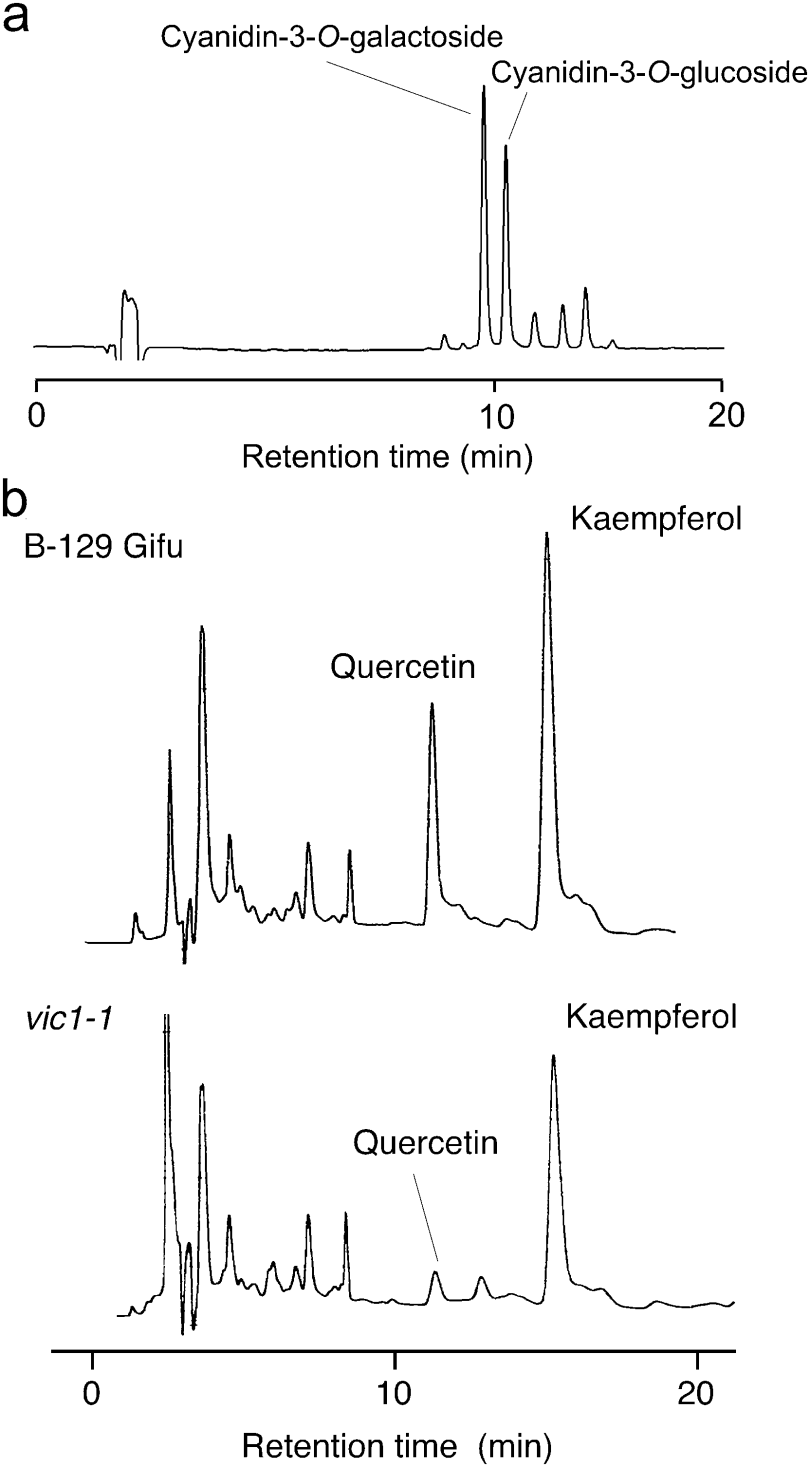
HPLC profiles of stem extracts of *L. japonicus.* **a** Anthocyanins extracted from B-129. HPLC was monitored at 520 nm. **b** Flavonoid aglycones isolated from B-129 and *vic1-1* (a class 1 mutant). Stem extracts with 80% (v/v) ethanol were hydrolysed with 3 M HCl at 100 °C for 2 h and analysed by HPLC at 236 nm

*Lotus japonicus* accession B-129 ‘Gifu’ accumulates red pigment in the petal, pedicel and stem (Fig. 2a, c, e). We detected two major and several minor anthocyanins in the acidified ethanol extract of stem segments by HPLC (Fig. 3a). Analysis of hydrolysed anthocyanins revealed that cyanidin was the major aglycone. Co-chromatography using authentic samples revealed that cyanidin-3-*O*-galactoside (idaein) and cyanidin-3-*O*-glucoside were the abundant anthocyanins in B-129 ‘Gifu’.

**Fig. 4:**
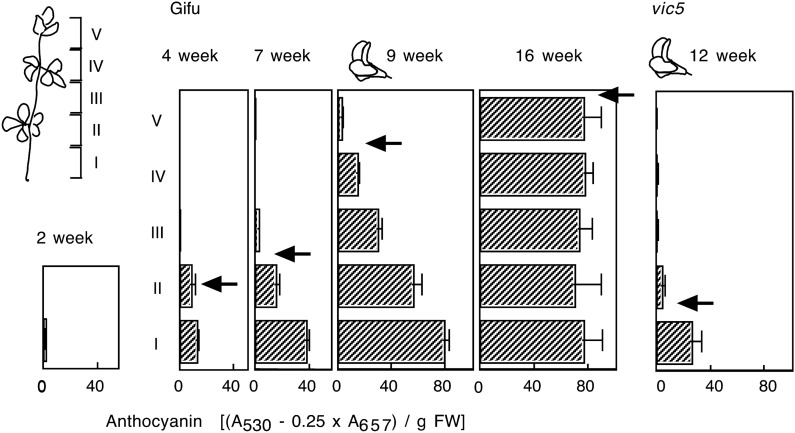
Stem anthocyanin content of *L. japonicus* plants grown in soil. Stems were cut into five parts and extracted with 1% HCl in methanol. Anthocyanin content was determined by colorimetry. The arrow shows the border of the visible pigmented area. The horizontal bar represents the standard error (SE; *n* = 3). Data of B-129 ‘Gifu’ and *vic5* (a class 2 mutant with lowered anthocyanin accumulation) are shown

To establish the criteria for the evaluation of mutants, we investigated anthocyanin accumulation in the stem of the standard accession B-129 ‘Gifu’. Stem cross-sections of B-129 plants cultivated in a greenhouse were obtained and further divided longitudinally into five. Anthocyanin concentrations were determined in these longitudinal sections. Figure 4 shows representative results using a standard cultivation condition. Anthocyanin first accumulated in two basal parts (I and II) at 2–7 weeks post-germination. At the flowering stage (9 weeks post-germination), anthocyanin accumulation was prominent in all four basal parts (I–IV), with the highest concentration in part I. At 16 weeks post-germination, the entire stem had accumulated a large amount of anthocyanin, which was almost the same as that in part I of 9-week-old plants. These results suggest several effective criteria for evaluating phenotypes of anthocyanin accumulation in the stem of *L. japonicus*. In cases where convenience is a priority, red or purple pigmentation can be a visual marker of anthocyanin accumulation in B-129 plants older than 4 weeks of age. Further unequivocal determination of the phenotype requires other criteria based on the developmental stage, instead of the period after germination, since plant growth and development are influenced by cultivation conditions, such as nutrition, temperature and light. Results shown in Fig. 4 and other independent observations suggest that the anthocyanin concentration in part I of the stem and the extent of pigmentation at the flowering stage would serve as excellent markers of anthocyanin accumulation (Fig. 2e). Red pigmentation, which appeared as red streaks on the standard petal and pedicel (Fig. 2a, c), serve as additional criteria.

**Fig. 5:**
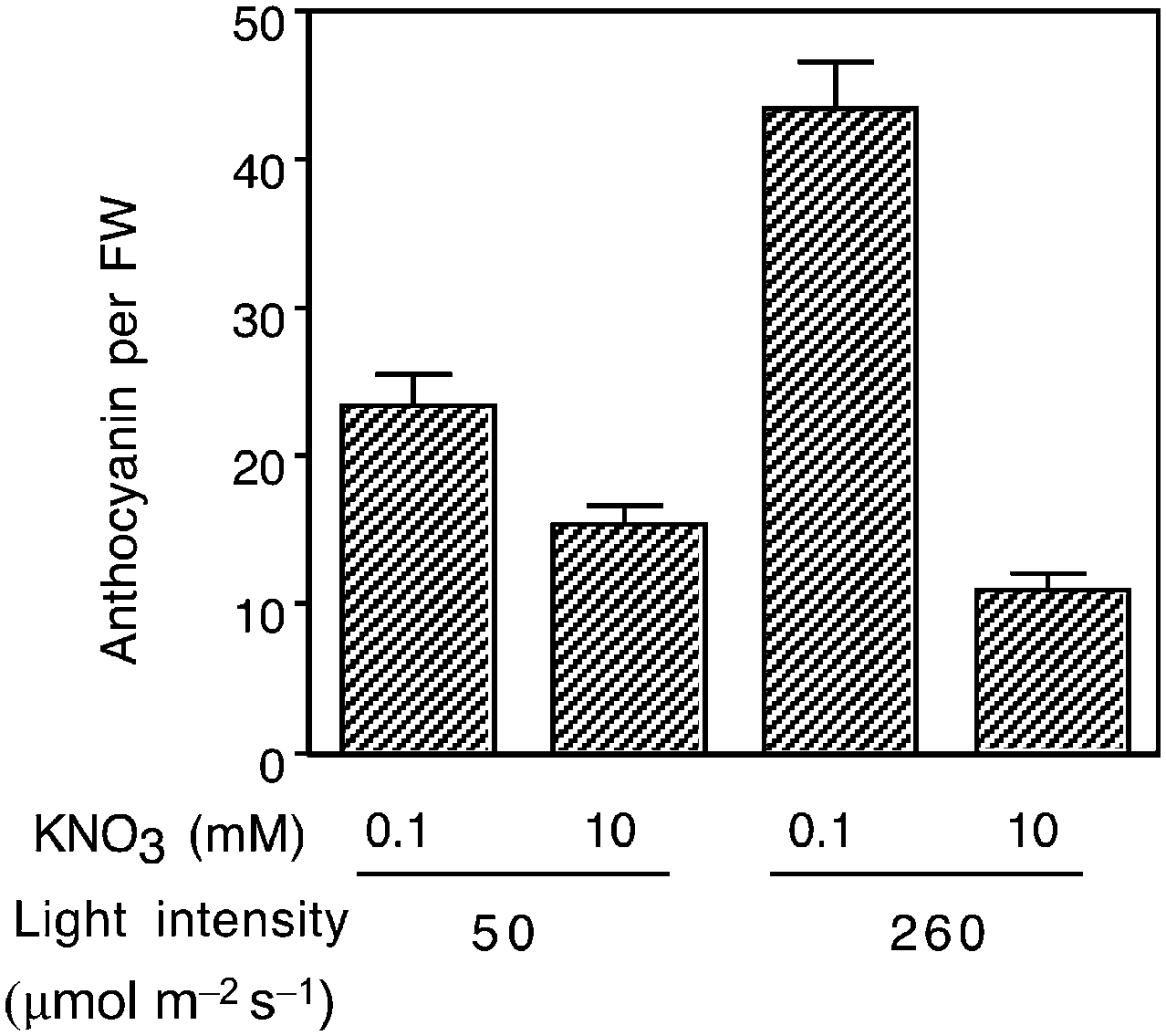
Anthocyanin content in *L. japonicus* B-129 seedlings. Sterilized seeds were sown on B&D agar media containing indicated concentrations of KNO_3_ and cultivated for 4 weeks. The vertical bar shows the SE (*n* = 8)

The accelerated accumulation of red pigments in the stem of mutants deficient in symbiotic nitrogen fixation (Schauser et al. 1999) implied the significance of nitrogen in the regulation of stem anthocyanin biosynthesis. To determine the effect of nitrogen on anthocyanin biosynthesis, B-129 plants were grown on B&D agar medium supplemented with permissive (10 mM) or limiting (0.1 mM) concentrations of KNO_3_ for 4 weeks. Anthocyanin accumulation was markedly enhanced under the nitrogen-limiting condition, particularly under high light intensity (260 µmol photons m^−2^ s^−1^) (Fig. 5). These results suggest that cultivation under nitrogen-deficient conditions enhances red pigmentation in wild-type stems, thus making the difference between wild and mutant phenotypes more apparent.

The M_2_ progenies of EMS-treated seeds were screened for anthocyanin-deficient mutants at two stages: first at 4–8 weeks post-germination and then at the flowering stage. Ten putative mutant lines were selected. After the confirmation of mutant traits and normal development in the M_3_ generation, five mutant lines with strikingly low anthocyanin concentration were selected. A similar approach was used to screen wild accessions collected from several areas in Japan, and three accessions (MG-13, MG-17 and MG-20) with low anthocyanin accumulation were selected (Table 1).

Loci involved in the stem anthocyanin accumulation were named *VIRIDICAULIS* (*VIC*) for the green (*viridi-*) stem (*caulis*). Table 2 summarizes the *vic* mutants identified so far. Genetic analysis by backcrossing with B-129 ‘Gifu’ revealed that five EMS-induced mutants and two wild accessions (MG-13 and MG-20) showed monogenic recessive inheritance (Table 2). On the other hand, anthocyanin accumulation in one wild accession, MG-17, was suggested to be regulated by two loci, based on the 15:1 segregation ratio observed in the F_2_ generation (Table 2). Mutants and natural variants were divided into two classes, according to the degree of anthocyanin deficiency. Phenotypes in class 1 were characterized by complete anthocyanin deficiency in the stem and standard petal (Ant^−^; Fig. 2b, e). On the other hand, phenotypes in class 2 accumulated anthocyanins to some extent, but the concentrations in the stem are distinctly low (Ant^±^) compared with the wild type B-129. Complementation tests of class 1 genotypes revealed four loci (*vic1*–*vic4*). Furthermore, complementation tests across class 1 and 2 genotypes revealed that MG-20 is a new allele of *vic4* and the Ant^±^ mutant (which was isolated from EMS-mutagenized lines) represents a new locus (data not shown). Based on these results, MG-20 and the Ant^±^ mutant were named as *vic4-2* and *vic5*, respectively. Anthocyanin deficiency in the stem of a representative class 2 mutant, *vic5*, at the flowering stage is shown in Fig. 4. The difference between B-129 and *vic5* was evident based on the above-described criteria, i.e. anthocyanin concentration in part I and the distribution of anthocyanin (Fig. 4). Two Ant^±^ class 2 lines, *vic4-2* and *vic5*, showed slightly red streaks on the standard petal, while the other Ant^±^ class 2 lines (MG-15 ‘Bishamon’, MG-17 ‘Mishima’ and MG-58 ‘Nichinan’) showed clear streaks, similar to the petal of B-129 ‘Gifu’ (Table 3). Red pigmentation in the pedicel was absent in all class 1 genotypes (Fig. 2d) and in Ant^±^ class 2 mutant and wild accessions, except MG-15 (Table 3). Severe nutrient limitation and long-term cultivation slightly accelerated the stem pigmentation in Ant^±^ lines but had no effect on anthocyanin accumulation in Ant^−^ lines (data not shown).

**Table 2:** List of anthocyanin-deficient mutants of *L. japonicus*

Locus	Origin^a^	Phenotype^b^	Backcross segregation^c^ N: M	Expected ratio	χ^2 d^
Class 1 (completely anthocyanin deficient)					
*vic1-1*	EMS	Ant^−^ Tan^−^	140: 49	3: 1	0.086
*vic1-2*	EMS	Ant^−^ Tan^−^	143: 43	3: 1	0.351
*vic2*	EMS	Ant^−^ Tan^−^	69: 21	3: 1	0.133
*vic3*	MG-13	Ant^−^ Tan^+^	133: 46	3: 1	0.046
*vic4-1*	EMS	Ant^−^ Tan^+^	185: 62	3: 1	0.001
Class 2 (lowered anthocyanin)					
*vic5*	EMS	Ant^±^ Tan^+^	122: 34	3: 1	0.854
*vic4-2*	MG-20	Ant^±^ Tan^+^	111: 39	3: 1	0.080
*vic*^e^	MG-17	Ant^±^ Tan^+^	208: 18	15: 1	1.133

^a^*EMS* mutagenesis by EMS treatment of seeds. See Table 1 for the accessions

^b^*Ant* anthocyanin accumulation, *Tan* condensed tannin (polymerized proanthocyanidins) accumulation. The standard wild type B-129 ‘Gifu’ is Ant^+^ Tan^+^

^c^*N* normal phenotype, *M* mutant phenotype, number of each phenotype observed in the F_2_ generation is shown

^d^A value of less than 3.8 indicates that the segregation is not significantly different from the expected ratio (*p* > 0.05)

^e^Unidentified loci

**Table 3:** Phenotype of flavonoid biosynthetic *L. japonicus* mutants

Line	Anthocyanin				Condensed tannin	Quercetin (Stem)
	Stem	Petal	Pedicel	Pod and Seed		
B-129	+	+	+	Brown	+	++
*vic1-1*	−	−	−	Yellow	−	+
*vic1-2*	−	−	−	Yellow	−	+
*vic2*	−	−	−	Yellow	−	+
*vic3*	−	−	−	Brown	+	+
*vic4-1*	−	−	−	Brown	+	+
*vic4-2*	−/+	–/+	−	Brown	+	N. D.
*vic5*	−/+	–/+	−	Brown	+	N. D.
MG-17	−/+	+	−	Brown	+	N. D.
MG-15	−/+	+	+	Brown	+	N. D.
MG-58	−/+	+	−	Brown	+	N. D.

*N. D.* not determined

Anthocyanins are biosynthesized via the phenylpropanoid/flavonoid pathway. The composition of related flavonoids can be used to characterize anthocyanin biosynthesis mutants. HPLC analysis after hydrolysis of aqueous ethanol extract revealed that two flavonols, kaempferol and quercetin, were the major soluble non-anthocyanin flavonoid aglycones in the stem of B-129 ‘Gifu’. A class 1 mutant, *vic1-1*, also contained both flavonols although the level of quercetin was relatively low (Fig. 3b). Flavonol concentrations in other class 1 mutants were highly similar (data not shown). These results suggest that in all class 1 mutants obtained, the mutant genes did not significantly affect the flavonoid pathway prior to the biosynthesis of dihydroflavonols (Fig. 1).

Proanthocyanidins are another class related to anthocyanins in the metabolic pathway (Fig. 1). In this study, polymerized proanthocyanidins (condensed tannins) were analysed by conversion to red anthocyanidins by heating with HCl in *n*-butanol. Figure 2g shows the presence of proanthocyanidins in the stem of B-129 and two class 1 mutants (*vic3* and *vic4-1*) and its absence in *vic1-1*, *vic1-2* and *vic2*. The three procyanidin-deficient mutants set yellow seeds and pods (Fig. 2f), whereas *vic3* and *vic4* presented a wild-type phenotype (brown pods and seeds) (Table 3). Strong genetic linkage of the three traits, no anthocyanin, no proanthocyanidin, and yellow seeds in the Ant^−^Tan^–^ mutants (Table 1) was suggested by the observation that more than ten F_2_ plants of no anthocyanin also lacked proanthocyanidins and set yellow pods and that no recombinant phenotype (e.g., no anthocyanin and brown seeds) was observed.

Next, nodule formation on the roots of Ant^−^Tan^−^ mutants was compared with that on the roots of B-129 plants using a domestic strain of *M. loti* (Tono). However, no significant difference was detected in the number, size and appearance of root nodules between Ant^−^Tan^–^ mutants and B-129 (data not shown).

## Discussion

*Arabidopsis thaliana* is the first model plant of the post-genomic era, and *L. japonicus* and *Medicago truncatula* are established as the major model of the Leguminosae family. This study was started in the last century at a very early stage of the research history of *L. japonicus* as a model organism, when information on the flavonoid composition of *L. japonicus* was limited (Morris et al. 1993; Nakaoki et al. 1956). Subsequently, metabolic profiling of *L. japonicus* by liquid chromatography Fourier transform ion cyclotron resonance mass spectrometry (LC-FTICR/MS) revealed ca. 60 flavonoids, mainly comprising flavonol glycosides and anthocyanins including cyanidin 3-*O*-galactoside and cyanidin-3-*O*-glucoside reported in this study (Suzuki et al. 2008). In addition, comparison of B-129 and MG-20 confirmed that B-129 stem contains cyanidin- and peonidin-based anthocyanins, whereas MG-20 stem contains no anthocyanin (Suzuki et al. 2008). Also, the tandem mass spectral database was helpful in connecting the metabolic quantitative trait loci (QTLs) of *L. japonicus* with individual flavonoid components (Sawada et al. 2012).

During the past 2 decades, progress in the research on the biosynthesis of isoflavonoids, which are characteristically dominant in the Leguminosae, has been documented using *L. japonicus* and other bioresources of this family. Elicitation of *L. japonicus* seedlings by reduced glutathione demonstrated the occurrence of vestitol, an isoflavan phytoalexin (Shimada et al. 2000). The enzymes of the vestitol biosynthesis pathway have been completely clarified, and the corresponding genes have been identified (Akashi et al. 2003, 2005, 2006; Shimamura et al. 2007; Uchida et al. 2017). Identification of the multigene families of the flavonoid/isoflavonoid biosynthetic enzymes in *L. japonicus* and their expression analysis upon elicitation and/or rhizobial infection have yielded important clues on the evolution of specific biosynthetic pathways and active and selective responses to biological and environmental stimuli (Akashi et al. 2006; Shimada et al. 2003, 2005, 2007).

Besides isoflavonoids, three related flavonoid classes of legumes, namely, anthocyanins, proanthocyanidins and flavonols, have gained significant attention for their role in plant–environment interactions (see below) and their exact biosynthetic pathway and mechanisms of metabolic control. For example, flavonoids have been shown to be important for nodule organogenesis; flavonols and isoflavones/flavones function as auxin transport inhibitors and rhizobial *nod* gene inducers, respectively (Subramanian et al. 2006; Wasson et al. 2006; Zhang et al. 2009). Despite the advances in our understanding of the biosynthetic relationship of anthocyanins and proanthocyanidins, the mechanism regulating the biosynthesis of oligo- and polymeric proanthocyanidins remains elusive. In *L. japonicus*, while the red stem colour and red streaks on the standard petal have been noted (de Nettangourt and Grant 1964; Grant et al. 1962; Schauser et al. 1999), these have not been sufficiently investigated either chemically or physiologically.

In this study, the criteria for screening and evaluating *L. japonicus* mutants defective in stem anthocyanin accumulation were developed based on the spatiotemporal characteristics of anthocyanin accumulation. These characteristics were identified using plants grown in a greenhouse for up to 12 weeks or using seedlings grown on agar media in vitro. The characteristics identified were as follows: (1) anthocyanin accumulation in the stem increased with the developmental stage or cultivation period, (2) extended from the lower part to the upper parts and (3) was enhanced by nitrogen limitation and intense illumination (Figs. 2 and 4). It has been reported that nitrogen availability can alter flavonoid biosynthesis in several plant species (McClure 1975), particularly in vegetative organs such as the root and stem (Bongue-Bartelsman and Phillips 1995; Cho and Harper 1991a, b; Coronado et al. 1995; Lea et al. 2007). Light-dependent anthocyanin production is a typical photoresponse, which is controlled by signals from photoreceptors (Fankhauser and Chory 1997). Anthocyanin accumulation in vegetative organs can be used as a visual marker for the genetic and physiological analysis of the signalling of environmental stimuli.

Artificially induced mutants and wild accessions of *L. japonicus* were categorized into three groups (Ant^−^Tan^−^, Ant^−^Tan^+^ and Ant^±^), according to flavonoid deficiency (Table 2). A barley (*Hordeum vulgare*) anthocyanin mutant, *ant-13*, deficient in proanthocyanidins showed extreme susceptibility to powdery mildew (von Wettstein et al. 1977), which suggests that proanthocyanidins are involved in plant defence. *Lotus* mutants lacking proanthocyanidins (*vic1* and *vic2*) should serve as a useful system for testing the participation of proanthocyanidins in the interaction of the host with herbivorous pests, pathogenic microorganisms and nitrogen-fixing symbionts. In this study, nodule formation in Tan^−^ mutants showed no difference from that in the wild type (B-129) upon inoculation with a domestic strain of *M. loti* (Tono). However, in right of the reported case that even the same *L. japonicus* mutant shows a different nodulation phenotype depending on the inoculated rhizobial strains (Shimoda et al. 2020; Yamaya-Ito et al. 2018), the effect of *vic* mutations on nodulation should be tested further using various *M. loti* strains and non-symbiotic rhizobia of *L. japonicus.* On the other hand, the interaction with rhizosphere microbiota via root exudates has attracted a great deal of attention in recent years. The effect of *vic* mutants on the rhizosphere microbiota would also be an interesting issue (Okutani et al. 2020; Sugiyama and Yazaki 2014; Suseela et al. 2016; Zhalnina et al. 2018).

Genetic analysis of Ant^−^Tan^−^ mutants of *L. japonicus* (*vic1* and *vic2*) indicated that at least two loci are essential for anthocyanin and proanthocyanidin biosynthesis. The presence of quercetin (Fig. 3b) in these mutants suggests that the biosynthetic pathway is functional up to the production of dihydroflavonol (Fig. 1). The simplest hypothesis is that the biosynthesis in the Ant^−^Tan^−^ mutants is blocked at the step producing leucoanthocyanidins (flavan-3,4-diols) from dihydroflavonols, which is catalysed by dihydroflavonol 4-reductase (DFR). The other class 1 mutants, *vic3* and *vic4*, contained polymerized proanthocyanidins (Fig. 2g). Genetic blocking of biosynthesis is likely to occur at the synthesis of cyanidin by leucoanthocyanidin dioxygenase (LDOX) (Saito et al. 1999), also known as anthocyanidin synthase (ANS). The other possibility is that *VIC* genes control several structural genes for anthocyanin/proanthocyanidin biosynthesis.

Well-investigated Arabidopsis loci essential for flavonoid biosynthesis are those collectively named as *TRANSPARENT TESTA* (*TT*) and *TRANSPARENT TESTA GLABROUS* (*TTG*), based on the reduced or abolished pigmentation in the seed coat of *tt* and *ttg* mutants (Appelhagen et al. 2014; Saito et al. 2013; Winkel-Shirley et al. 1995). Seed coat pigments are thought to be related to the proanthocyanidin pathway (Abrahams et al. 2002). Some *TT* genes encode biosynthetic enzymes (Chang et al. 1988; Feinbaum and Ausbel 1988; Winkel-Shirley et al. 1992) and regulatory factors (Nesi et al. 2000; Walker et al. 1999). The seed colour of *L. japonicus* Ant^−^Tan^−^ mutants was similar to that of Arabidopsis *tt* mutants, but only limited variation of Ant^−^Tan^−^mutants has been identified so far. Further screening would facilitate the identification of mutants defective in other steps of anthocyanin biosynthesis because the scale of screening in this study (14,000 M_2_ seeds) was far smaller than that expected to saturate the loci essential for anthocyanin accumulation. However, we could not exclude the possibility that structural and regulatory genes involved in some steps of the pathway exhibit redundant functions in *L. japonicus* (Fig. 1) or that mutants defective in the early steps are sterile or lethal. It is noteworthy that the *TT2* homologs of *L. japonicus* contribute to proanthocyanidin accumulation mediated by anthocyanidin reductase (ANR) (see below; Yoshida et al. 2008). Moreover, LjTT2 (R2R2-MYB protein), LjTT8 (bHLH protein) and LjTTG1 (WDR protein) form a ternary transcription complex that acts on the promoter of *LjDFR2*, one of the five paralogous *DFR* genes of *L. japonicus* (Shimada et al. 2005), leading to proanthocyanidin biosynthesis, while the substitution of LjTT2 in the ternary complex with LjPAP leads to the biosynthesis of anthocyanins (Yoshida et al. 2010).

Stereoisomers of flavan-3-ols are intermediates for the biosynthesis of proanthocyanidin oligomers and produced through at least two pathways characterized by representative enzymes (Fig. 1). The activity of leucoanthocyanidin reductase (LAR) was detected in *Lotus* cell free extract (Skadhauge et al. 1997). The ANR enzyme was shown to be encoded by the *BANYULS* gene in Arabidopsis and its orthologs in *M. truncatula* (Devic et al. 1999; Xie et al. 2003) and *Lotus* spp. (Paolocci et al. 2007; Yoshida et al. 2008). In Arabidopsis seeds, the ANR pathway is probably a dominant pathway for proanthocyanidin biosynthesis because the *tt18* mutant, which lacks LDOX activity, had greatly reduced levels of proanthocyanidins (Xie et al. 2003). The phenotype of Ant^−^Tan^+^ mutants of *Lotus* spp., such as *vic3* and *vic4*, suggests that the LAR pathway, which is independent of anthocyanidin biosynthesis, may function as a bypass for the production of proanthocyanidins in *Lotus* stems. However, considering a new pathway into a flavan-3-ol stereoisomer reported for *M. truncatula* (Jun et al. 2018), detailed examination of the proanthocyanidin stereochemistry and the biosynthetic enzymes involved, possibly taking advantage of these *vic* mutants, may lead to important findings, which could help understand this complicated metabolic pathway.

In a previous study, 17 characteristics of 66 *L. japonicus* accessions collected from different regions of Japan were evaluated, and the correlation among traits and between traits and geographic conditions of the habitats were statistically analysed (Suginobu et al. 1988). Stem colour (anthocyanin content), leaf colour (chlorophyll content) and plant weight showed a significant correlation among each other and with the latitude. The correlation between the anthocyanin content and other physiological characteristics implies that genes partially involved in flavonoid biosynthesis are pleiotropic and perform physiological roles in environmental adaptation and/or growth and development.

The wild accessions of *L. japonicus* described in this study showed considerable variation in anthocyanin accumulation and inheritance of the mutant phenotype (Table 2). It is noteworthy that the collection sites of MG-13, MG-15 and MG-17 accessions were no more than 60 km away from each other. In some cases, wild accessions coexisted with other plants exhibiting the wild-type stem colour at the collection sites. These facts suggest marked diversity of anthocyanin accumulation in wild-grown *L. japonicus*, although the underlying reason is unknown. It is also unclear whether the variants are fully adapted to the environment of their habitat since the number of individuals with the mutant phenotype varied among the collection sites. Several plants of the MG-15, MG-20 and MG-58 accessions were clustered at the collection sites; however, only single plants of MG-13 and MG-17 were found at a given site. Thus, the frequency of *L. japonicus* individuals with a green stem also varied.

Numerous plants have been used in molecular and genetic studies of the flavonoid pathway. Several *L. japonicus* resources are now available for molecular genetics and genomics analyses (Asamizu et al. 2000a, b; Fukai et al. 2012; Hashiguchi et al. 2018; Hayashi et al. 2001; Kamal et al. 2020; Li et al. 2020; Nakamura et al. 2002; Pedrosa et al. 2002; Sandal et al. 2002; Sato et al. 2001, 2008). Fine mapping for cloning the *VIC* genes is currently under progress. *L. japonicus* is a model plant and represents another system especially suitable for exploring the regulation of anthocyanin biosynthesis in vegetative organs and the specific functions of flavonoids in leguminous plants.
