# Correction to: Mutants of Lotus *japonicus deficient* in flavonoid biosynthesis

**DOI:** 10.1007/s10265-021-01272-w

**Published:** 2021-02-24

**Authors:** Toshio Aoki, Masayoshi Kawaguchi, Haruko Imaizumi-Anraku, Shoichiro Akao, Shin-ichi Ayabe, Tomoyoshi Akashi

**Affiliations:** 1 Department of Applied Biological Sciences, Nihon University, Fujisawa, Kanagawa 252-0880 Japan; 2 Division of Symbiotic Systems, National Institute for Basic Biology, Okazaki, Aichi 444-8585 Japan; 3 Institute of Agrobiological Sciences, National Agriculture and Food Research Organization, Tsukuba, Ibaraki 305-8634 Japan

## Correction to: Journal of Plant Research https://doi.org/10.1007/s10265-021-01258-8

In the original publication of the article, the header row of Table 3 was incorrectly published and “tannin” and “Quercetin (Stem)” were placed under “Condensed”. The right most headers should read “Condensed tannin” and “Quercetin (Stem)”, respectively.

The original article has been updated.

